# Care-seeking strategies of migrants during the transition from a specific primary healthcare facility for uncovered individuals to common ambulatory general practice: A French qualitative study

**DOI:** 10.1186/s12889-024-19048-x

**Published:** 2024-06-10

**Authors:** Jego Maeva, Desrues Anne, Fall Marie, Janczewski Aurélie, Gentile Gaetan, Auquier Pascal, Tabélé Clémence, Khouani Jérémy

**Affiliations:** 1 Department of General Practice, Faculty of Medicine, Aix-Marseille Univ, 27 Bd Jean Moulin, 13385, Marseille, France; 2 Aix-Marseille Univ, UR3279 CERESS Marseille, France; 3 Department of Public Health, University Hospital APHM, Marseille, France; 4 UMR S 1106, Aix Marseille Univ, INSERM, INS, Inst NeurosciSyst, Marseille, France

**Keywords:** Delivery of healthcare, General practitioners, Underserved people, Primary care, Migrants, Accessibility to health services

## Abstract

**Background:**

Migrants have complex health needs but face multiple barriers to accessing health care. In France, permanent healthcare access offices (PASSs), as specific primary health care facilities (SPHCs), provide care to people without health insurance coverage. Once these patients obtain health insurance, they are referred to common ambulatory general practice. The aim of this study was to explore migrants’ experiences and strategies for seeking common primary care after having been treated by an SPHC.

**Methods:**

We conducted a qualitative study based on grounded theory between January and April 2022. We held semi-structured interviews with migrants who had consulted a PASS. Two researchers performed an inductive analysis.

**Results:**

We interviewed 12 migrants aged 22 to 65 to confirm data saturation. The interviewees relied on “referents”: professional referents (to be properly treated for specific health problems), guides (to find their way through the healthcare system), or practical referents (to address practical issues such as translation, travel needs, or medical matters). Those who considered the PASS to be a referent expressed disappointment and incomprehension at the time of discharge. Referral procedures and the first encounter with common ambulatory general practice were decisive in whether the interviewees accessed and stayed in a coordinated primary care pathway. The perceived quality of care depended on a feeling of being considered and listened to. For interviewees who received first-time services from an ambulatory general practice, the way in which they were referred to and their first experience with an ambulatory GP could influence their adherence to care.

**Conclusions:**

The conditions of transition from SPHCs to common ambulatory general practice can impact migrants’ adherence to a coordinated primary care pathway. Referral can improve these patients’ care pathways and ease the transition from a PASS to ambulatory care. Healthcare professionals at SPHCs should pay special attention to vulnerable migrants without previous experience in ambulatory general practice and who depend on referents in their care pathways. For these patients, adapted referral protocols with further individual support and empowerment should be considered.

**Supplementary Information:**

The online version contains supplementary material available at 10.1186/s12889-024-19048-x.

## Background

A lack of access to healthcare is a major concern for vulnerable people who face complex health and social needs [1, 2]. In 2015, the United Nations (UN) General Assembly adopted a resolution setting of 2030 as the target date for achieving universal health coverage (UHC) [3], in which “all people have access to the full range of quality health services they need, when and where they need them, without financial hardship.” [4] The World Health Organization (WHO) noted that providing financial protection for all individuals is essential for achieving UHC [5], which is not a reality today [6]. Among migrants, undocumented migrants and asylum seekers are particularly vulnerable; they face poor social conditions and have lower health status than the general population (a higher prevalence of chronic diseases,mental health issues, and poorer perceived health) [7–10]. Although vulnerable migrants have important and complex health needs [9, 11], they face barriers to accessing adequate and efficient healthcare, particularly because of cultural misunderstandings, language barriers, discrimination, negative care experiences, and financial barriers [1, 2, 12–15].

A recent European study revealed that immigrant-friendly healthcare policies were significantly associated with better health and fewer unmet needs for non-financial reasons [16]. If free healthcare for vulnerable people provides additional resources, creating dedicated care networks for people without health insurance coverage limits the establishment of equal and universal access to healthcare [6, 12, 13]. In France, all foreigners, regardless of their residency status, theoretically have the right to healthcare. The steady increase in migration is generating significant healthcare needs, which are mainly met by existing services such as healthcare access services (PASS) [17]. The PASS are specific primary health care facilities (SPHCs) that provide care to vulnerable people who do not have health insurance and therefore lack access to common primary care (either they have never had any health insurance, especially for newly arrived migrants, or their health insurance rights are violated). PASS are mostly located in hospitals and provide access to free healthcare (including consultations with general practitioners (GPs) or specialists, access to paraclinical examinations, and medication) for patients who need care until they receive or recover adequate health insurance. Patients visiting SPHCs are most often migrants and face social or health vulnerabilities in addition to their lack of health insurance (poor living conditions, homelessness, seeking asylum, undocumented immigration status, exposure to violence, etc.) [10, 18, 19]. As a result, in this paper, we use the term “vulnerable migrants” to refer to migrants without social health insurance who have visited PASS. Once they have acquired health insurance, vulnerable migrants visiting PASS are referred to common primary care, where they identify a GP and access a stable coordinated care pathway [20]. In the French healthcare system, ambulatory GPs have the tasks of the “médecin traitant” (treating physician), introduced by the health law of 2005; they are the gatekeepers of the healthcare system and provide global and coordinated care for patients. All patients in typical circumstances can declare that they have a treating physician to the French National Health System. Illegal migrants cannot do so but benefit from the same access to global coordinated care pathways through ambulatory GPs and often identify them as referral physicians [21]. GPs and their care organizations can enhance or limit vulnerable migrants’ access to needed care, depending on their ability to develop a culturally patient-centered approach and listen to their patients’ health and social needs, a welcoming environment, and the availability of interpretation services [22, 23].

In 2019, a US interventional study of a transitional care practice for vulnerable individuals who made appointments and sent hospital reports to community GPs revealed a significant reduction in hospital use during the 180 days following hospital discharge [24]. However, the transition from hospitals to community care can lead to a breakdown in patient care. French studies have reported the risk of breakdown and difficulties anchored in a primary care pathway after PASS discharge: 22% of patients did not consult a GP in the 3 months following discharge or referral to a GP [25], 30% did not consult a primary care physician for 12 months (in the year 2009, 1 year after PASS discharge), and only 45% declared that they had a treating physician 2 years after PASS discharge [26]. It is necessary to evaluate complementary strategies and healthcare organizations that could improve the integration of patients into a coordinated care pathway after patients are followed in SPHCs.

The PASS-MULTI study is a multicenter, randomized, open-label, comparative study conducted in Marseille (France) to assess the impact of a multidisciplinary PASS system, including a pharmaceutical interface in addition to social, medical, and healthcare teams on the 12-month re-hospitalization rate of patients in precarious situations. An ancillary study of the PASS-MULTI focused on evaluating a referral protocol from hospitals to common ambulatory general practice for patients following hospitalization [20]. The hospital-to-community protocol consisted of the following steps:

- a medical consultation in which the patients were given their medical records and prescriptions if needed.
- a pharmaceutical consultation, with information about the prescriptions and educational therapy.
- a specific transitional hospital-to-community consultation, with information on patient rights and healthcare organizations and the delivery of an informational booklet.

Depending on the patients’ needs, patients could search for and make an appointment with a GP by themselves, receive a list of GP contacts, have an appointment with a GP scheduled by a PASS professional, or receive individual support with health mediation for their first appointment with a GP [20].

Sixty patients were included in this study between November 2020 and August 2022, including 35 who benefited from the hospital-to-community protocol. Almost all of the patients (58 of 60) were migrants. Among them, 68.8% of those who benefited from the hospital-to-community protocol had consulted the GP they were referred to within six months, whereas 40% did not benefit from this protocol. Only 25% of the patients had declared a treating physician when they could. While 63.4% of the patients needed care, 42.3% had not consulted the GP they identified as a referent doctor [20].

We did not find any study focusing on the experience of care among vulnerable migrants during their transition from SPHCs to common primary care. From the perspective of UHC, integrating vulnerable migrants from a specific free healthcare system into the common primary care system involves exploring their care experiences and mechanisms for seeking care in these situations.

We aimed to elucidate migrants’ experiences and strategies for seeking care in common primary care after having been followed by a specific healthcare facility. We chose a qualitative approach to better understand complex issues such as patient perspectives and the logic behind healthcare use for underserved populations [27–29].

## Methods

### Study design

We conducted a qualitative study based on grounded theory from January to April 2022 among migrants who had consulted in a French SPHC, in particular the hospital-based PASS in Marseille.

### Population and sampling

The target population consisted of adult migrants without health insurance coverage who experienced a transition from dedicated services for vulnerable people to common ambulatory general practice after receiving health insurance.

Patients included in the PASS-MULTI study were older than 18 years, had recently been admitted to the hospital and needed treatment at baseline, were in a precarious situation (EPICES score ≥ 48) [30] and did not have full health insurance coverage at the beginning of care in the PASS.

We carried out our study on a cohort of migrants included in the PASS-MULTI study who had acquired full healthcare coverage. We included patients from both the interventional arm of the PASS-MULTI study (hospital-to-community protocol) and the non-interventional arm in this qualitative study. Patients who met the inclusion criteria for the PASS-MULTI received information on the study in their own language, and their consent to participate was sought by a professional outside the care team. A professional telephone interpretation service was used as needed to provide information to the participants in their native language, and they were offered the option to be accompanied by someone they trusted, particularly for those who could not read [20].

We used a purposeful variation sampling method and progressively targeted the inclusions to achieve a diversified sample on age, gender, language status (French-speaking or not), and social insurance. We also collected data on education level, housing status, duration in France and health status (having a chronic disease or not). We halted additional inclusions when saturation of the data was reached [29, 31, 32].

### Data collection

Two researchers performed semi-structured interviews between January and April 2022 (M.F., a resident in general practice and A.D., a sociologist), face-to-face at the PASS, or by telephone for patients with mobility issues. The interviews were audio-recorded and then fully transcribed. A professional interpretation telephone service (“Interprétariat Service Migrants,”, commonly used at the PASS for consultations with patients who need assistance understanding and speaking French) was used for non-French-speaking patients. The interview guide contained questions on experiences with healthcare at the PASS, the transition from the PASS to common ambulatory general practice, the perceived differences between the PASS and ambulatory care, patient use of care, patient opinions regarding common ambulatory general practice and patient care pathways in primary care. The guide was tested with one patient and modified based on the results to clarify emerging patterns as they became known. To improve reliability, the researchers used a “logbook” to record their emotions, the evolution of their assumptions, and their intellectual process for analysis [33].

### Analyses

We performed an inductive analysis based on grounded theory using Nvivo® 14. Two researchers (M.F. and A.D.) triangulated the analysis, supervised by a third researcher (M.J.), with regular discussions about the key stages of analysis. First, we analyzed verbatim transcripts of the patients’ interviews and highlighted meaningful sections. Second, we categorized similar themes and gave them the same codes (“properties”). Third, we clustered closely related codes (i.e., categories) [29, 31, 34].

### Ethical issues

We provided patients with a detailed explanation of the study’s focus (the aim, data recording, and use). We obtained oral informed consent to participate from all participants prior to inclusion. We anonymized all interviews as soon as they were held. The PASS-MULTI received authorization from the personal protection committees of Sud-Ouest and Outre-Mer I (approval #2019-A02740-57) and from the French data protection authority (CNIL) (registration #DR-2021–143).

## Results

We contacted 41 patients included in the PASS MULTI study. Among them, 14 were unreachable (voicemail, wrong number), 2 refused to participate in the qualitative study (because they did not recall having used the PASS), 2 did not appear at the meeting, and 11 gave formal consent but ultimately did not take part. We obtained data saturation at the 11th interview, validated by the 12th. The interviews lasted an average of 56 min (24 to 80 min). All but one (the 12th interview, conducted by phone) were performed face-to-face at the PASS office. The patients were 22 to 65 years old (median age: 40 years). Eight of the 12 patients were men. Seven patients were from sub-Saharan Africa, 3 were from North Africa, and 2 were from Eastern Europe. The housing status of 11 patients was known: Only 3 patients had stable housing (6 lived with friends or family, 1 lived in a shelter, and 1 was living rough). All of the patients had access to common primary care: Eight patients were in an irregular situation and obtained free medical aid (state medical aid) after PASS discharge; most of the others had obtained universal free complementary health coverage. Eight patients were from the interventional arm and three were from the non-interventional arm. Three patients did not speak French. First, the interviews were held in English with a researcher who spoke English; second, a professional phone interpretation service was used (Table 1).

**Table 1:** Migrant characteristics

N	Interview duration (minutes)	Age	Gender	Beneficiated from referral protocol	Administrative status	Health insurance	Housing status	Geographic origin and duration in France	French-speaking	Education level	Chronic disease
1	24	40–50	Man	No	Visa	UHC (without complementary health insurance)	Stable housing	Algeria ≥ 20 years	Yes	Secondary school	Yes
2	50	50–60	Man	Yes	Irregular situation	State medical aid	Insecure (with friends)	Bulgaria 1 to 5 years	No (professional telephone interpretation service)	Secondary school	Yes
3	45	30–40	Woman	Yes	Irregular situation	State medical aid	Insecure (with friends)	Nigeria 5 to 10 years	No (English-speaking with researcher)	Secondary school	Yes
4	60	60–70	Man	Yes	Irregular situation	State medical aid	Insecure (with friends)	Algeria ≥ 20 years	Yes	Secondary school	No
5	60	20–30	Man	Yes	Irregular situation	State medical aid	Unknown	Senegal 1 to 5 years	Yes	Primary school	No
6	50	30–40	Woman	Yes	Irregular situation	State medical aid	Insecure (with family)	Comoros 1 to 5 years	Yes	Secondary school	No
7	50	30–40	Woman	Yes	Irregular situation	State medical aid	Insecure (with friends)	Comoros 5 to 10 years	Yes	Higher	No
8	70	40–50	Woman	Yes	Irregular situation	State medical aid	Houseless (at a shelter)	Cape Verde ≥ 20 years	Yes	Higher	Yes
9	60	20–30	Man	Yes	Visa	UHC with free complementary health insurance	Stable housing	Comoros ≤ 1 year	Yes	Higher	Yes
10	80	30–40	Man	No	Irregular situation	State medical aid	Roofless (on the street)	Algeria 5 to 10 years	No (professional telephone interpretation service)	Secondary school	Yes
11	62	40–50	Man	Yes	Resident permit	UHC with private complementary health insurance	Stable housing	Cape Verde 5 to 10 years	Yes	Secondary school	No
12	58	30–40	Man	No	Resident permit	UHC without complementary health insurance	Insecure (with friends)	Armenia ≥ 20 years	Yes	Secondary School	No

**Fig. 1:**
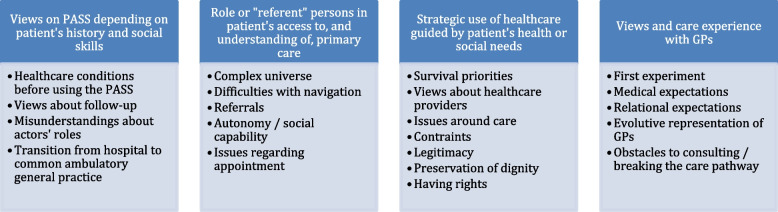
Coding tree with the main categories identified via inductive analysis

Four categories emerged from the analyses: (1) heterogeneous views on the PASS, which varied based on patients’ history and social skills; (2) the role of “referent” persons in patients accessing and understanding primary care; (3) the strategic use of healthcare guided by patients’ health or social needs; and (4) views about ambulatory GPs’ and patients’ anchoring in a coordinated care pathway (Fig. 1).

### Heterogenous views about PASS services relying on patient history and social skills

Patients followed in the PASS had lived in France for various lengths of time and had diverse registration statuses in the healthcare system and external referrals. We identified three distinct views directly linked to the patients’ prior resources:

- The “Anonymous” PASS: Care was brief or non-existent, and patients were autonomous and already involved in a care program. The PASS represented a temporary break in healthcare availability and was not identified as a benchmark structure. Its impact on their care was perceived as minimal. The end of PASS care meant returning to normal care.
- The “transitional” PASS: Patients had other referents and resources to guide them through the healthcare system. Patient adherence to the mainstream system depended more on the other referents than on the PASS.
- PASS as a reference: Patients received long-term follow-up at the PASS and had few or no other referents. The PASS represented access to the French healthcare system. Dependence on the PASS was high, and transitions put their continued care at risk. The PASS could have been a reference for one of the three patients from the non-interventional arm: this patient was homeless, was being treated for cancer and could not find any help (the 10th interview). The 2 other migrants in the non-interventional arm identified the PASS as transitional. Of the four patients who had lived in France for more than 20 years, only two considered the PASS to be anonymous. The PASS was a reference for the other two patients due to the precarity of their housing and legal status in France.

Patients identified the PASS as a tailored service for migrants and were reliant on and grateful for access to the program. The multidimensional care provided and the follow-up, which they perceived as organized, reassured them and gave them a sense of having received high-quality care. They perceived the professionals as empathetic and attentive to their needs. *“Here, it is as if they’re programmed to serve you. It is different. You come in, and you feel at ease. […]We give you everything. The medication…”* (the 11th interview).

Their understanding of the PASS was vague, and professionals’ attitudes influenced patients’ perceptions of the quality of care received. “*Yes, you take a lot more time to explain things. Some doctors [mimes someone doing things veryquickly], and that's it, bye*” (interview 3).

### Role of referent persons in patients accessing and understanding primary care

Patients perceived the healthcare system as a complex universe and experienced difficulties understanding and navigating it. They felt shunted from place to place and did not always understand the objectives of each encounter or the role of the person with whom they were speaking (doctors, nurses, health mediators, and social workers in the PASS): *“No, I didn't understand a thing, I just did as they told me. They told me to go to [name of hospital] and this and that. I did as they told me”* (the 10th interview).

They were discouraged by the numbers of people to whom they had to talk: *“I don't know who to go and see, and they often suggest a lot of people, but I'm ashamed to go and see them, so I don't know who to go and see, because there are so many […] I wanted to see that one person who knows everything”* (the 12th interview).

To navigate the healthcare system, patients relied on referents, which were individuals or structures. We identified three referral functions:

- The **“expert” referent,** a professional that personalized care and who was identified as a trusted interlocutor for specific health problems*, said, “Since I've been at the PASS, you've all been very good at looking after patients with high sugar levels”* (the 3rd interview).
- The **“guide,”** who was the gateway to the care system and helped to solve problems. Patients mostly relied on social workers or primary healthcare professionals. *“I spoke to my midwife yesterday, and I told her I hadn’t done the tests because I didn't have the money, my state medical aid isn't there yet… he called there… […] he told me he'd keep me informed”* (the 7th interview).
- The **“practical referent,”** a close relative with more abilities. He/she was able to use his/her own resources to overcome obstacles in accessing care (e.g., translation, travel, dealing with discrimination, etc.). Having visited an emergency room three times without success, the 10th patient stated: *“I had a friend whose car I repaired … a French man, someoneFrench-speaking, […] he took me to the hospital and spoke with the staff […] And after this exchange with this friend, the members of the hospital [place] redirected me to [hospital name] and there I was diagnosed with cancer.”*

Even if the referents could help patients navigate the system, some migrants needed more time to understand the care system and gain autonomy. When they did not acquire a good understanding of the system and stayed dependent on referents, they could be more vulnerable in their pathways to care. Referents’ support could end abruptly, leading to a breakdown in care. *“I had this person […] she followed me and then, I don't know, suddenly, she said […] "well, I can't follow you anymore". I said "ah, you can't replace me"; she said "no, no, you'll have to look at something else,’ so I gave up”* (the 12th interview).

Patients who considered the PASS as a referent or who were dependent on their care pathway expressed disappointment and sometimes incomprehension at the end of the PASS: *“I don't have any explanations, but I think they've finished their work with me”* (the 2nd interview). Referral procedures and the first encounter with common ambulatory primary healthcare were decisive for patients accessing and staying in a coordinated primary care pathway. For example, a patient who had been given a list of doctors did not make an appointment: “*Yes, they said that I had to be followed up by a doctor, and they gave me a list of these doctors that I could perhaps go and see… but I didn't go and see [them]…”* (the 9th interview).

### Guidance of patients’ strategic use of healthcare for their health and social needs

Strategies for seeking care were guided by three unique approacheswhich affected the type of care used (e.g., GP , emergency department, etc.):

- **Instrumentalist logic**: With priorities focused on survival, the body became a tool. Then, care was evaluated according to its ability to address survival priorities: “*Yes, now I’m starting to walk more than before; I said ‘forget it, I’m happy” *(the 8^th^ interview).Patients’ perceived need for care and follow-up could therefore differ greatly from a medical perspective.
- **Logic of efficiency**: As patients have daily constraints, care must be both medically effective and practical (e.g., proximity, consultation time, cost, simple access, etc.).

“*There are a lot of people. You had to wait. Otherwise, you'll go early in the morning, but I can't get there at 6. [...] but now it's[has] already been more than a year that I [have] not [gone] there... because of that. [...] When I’m sick, I go to the emergency room” *(the 7^th^ interview).

- **Strategies to preserve dignity:** Patients could sense the effects on their dignity, particularly in cases of discrimination. Despite the direct health benefits of care, patients could avoid or abandon it after experiencing misunderstandings or other negative experiences. They noted that preserving their dignity was a primary consideration. For example, in the 11^th^ interview, the interviewer asked:* “Did you feel a difference...?” *The patient replied, “*Yes, as I’m a foreigner... I felt that. In addition, [at] the second appointment… , I said ‘No, I’m not going. I'm not going.’ Because I felt the difference of not being French*...”

### Views about ambulatory GPs and patient adhesion to ambulatory care

Patients’ views about common ambulatory general practice were formed by their experiences and their reasons for seeking care; they expressed relational expectations, and their perceived quality of care depended on a feeling of being considered and listened to. These limited avoidance strategies were linked to feelings of discrimination. *“In the way he communicates, he makes you feel at ease. Because sometimes that is what you need, to feel at ease*” (the 11th interview).

For patients who began receiving care at an ambulatory general practice, the first experience with a GP seemed decisive for their adherence to care. For example, a patient referred to a doctor whom the patient felt was neglectful never returned to outpatient general practice. When patients had long-term follow-up care with ambulatory GPs, they expressed more expectations linked to the GPs’ care skills (e.g., follow-up, prescribing medication, making a referral, a clinical examination, etc.). After a negative experience, these patients were more likely to consider changing GPs than abandoning outpatient medicine entirely.

The practical organization of reception and care was important for patient adhesion to care. The ability to access care with or without an appointment was a recurrent issue and illustrated the perceived complexity of seeking care as well as the patients’ use of cure-related logic. However, the appointment represented symbolic acceptance in the French healthcare system: “*My GP used to work without appointments, but now with appointments and everything, and he accepted me*” (the 1st interview).

Patients were reassured by organized medical follow-up. However, the appointment process was viewed as difficult. The anticipation created by the appointment conflicted with instrumental logic and an urgent approach to treatment: *“He gave us another appointment. I’m not going to make another appointment. Because I’m not used to going to see him all the time. I go to see him when I’m sick*” (the 6th interview). The appointment induced social expectations of commitment, punctuality, and self-presentation that were difficult to fulfill. *“I cannot commit myself… every time I have nothing, and I cannot. Sometimes I shave badly; I cannot shave because I do not have a place and I’m not changed sometimes [after] 3 or 4 days*” (the 12th interview). The constraint of appointments led to breaks in care, with patients losing contact with their GPs or changing their strategies to seek care. *“I went there, and they told me she was not there. I do not know what to do. […] I missed the appointment because I had other appointments at the same time*” (the 3rd interview).

## Discussion

### Main results and discussion

**Fig. 2:**
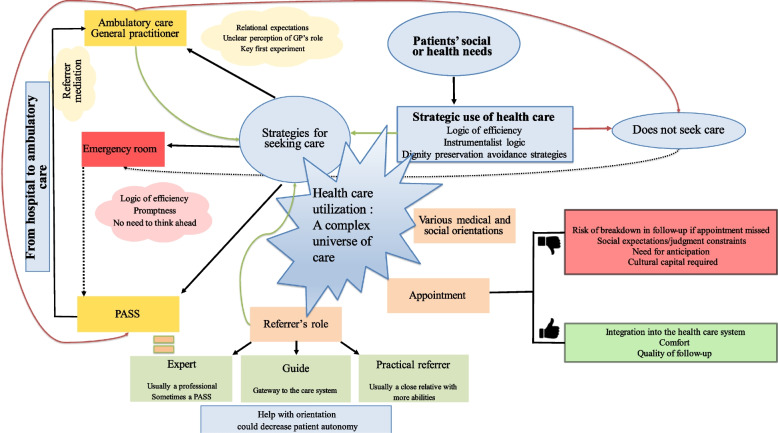
Determinants and strategies for seeking care during the transition from a French hospital with free healthcare access office to a common ambulatory general practice

After receiving care in specific healthcare facilities such as PASS, migrants’ adherence to a coordinated primary care pathway through an ambulatory GP seemed to depend on how the transition from the hospital to ambulatory services was handled. Migrants in this situation had specific needs concerning conditions for accessing primary care, as highlighted by Levesque’s conceptual framework for accessing care. They had both health and social needs, and their use of healthcare was strategic. Their perceived quality of care depended on a feeling of being considered and listened to. They experienced difficulties in using and navigating the healthcare system, which they perceived as a complex universe. The presence of a referent to guide them through these processes could facilitate a patient’s care pathway and transition from the PASS to ambulatory care. However, migrants who relied on referents and did not have personal skills to navigate the healthcare system were more vulnerable in their care pathways. When they lost the referent, their care pathway broke down [35] (Fig. 2).

The need for referents to access primary care has been noted for vulnerable migrants, such as undocumented migrants or asylum seekers. In a 2017 qualitative study in Marseille, undocumented migrants accessed primary healthcare through a specific social network (including associations, community links, and social workers) [21]. Other studies have shown the need for support to navigate the healthcare system, which could be enhanced by community or health mediators [36, 37]. Our results showed that for vulnerable migrants, the SPHC itself could become a referent. In the PASS-MULTI study, more than one of 3 patients had not consulted a GP within 6 months after the end of their PASS follow-up. A referral protocol significantly improved their probability of consulting an outpatient GP in the 6 months following PASS discharge, but more than 30% still did not [20]. In our study, the patients had various views about SPHCs and strategies for seeking care, depending on their prior resources and experience in ambulatory general practice. Patients for whom the PASS was a referent seemed more dependent on referents to navigate and use the common primary care system. Patients could feel more positive about care experiences and health service uptake in culturally appropriate care or specific migrant-friendly health services [38]. However, accessing a specialized care network could enhance feelings of exclusion and stigmatize care [39]. For the most vulnerable patients, during the transition from a PASS to common ambulatory general practice, a referral protocol using health mediation to improve initial contact with an ambulatory GP could resolve this contradiction. Longer individual support and complementary empowerment approaches should also be evaluated.

The patients’ first experience with a GP seemed to determine their strategies for seeking care. The practical organization of care provided by practitioners was important for patients’ adherence to common ambulatory general practice. For migrants who experienced a transition from hospital to community care, the care provided had to be easy to access and use. In the opposite case, migrants used referents to navigate their care pathway. A 2014 Dutch study highlighted the importance of the first contact with GPs, with undocumented migrants’ expectations focusing on the relationship and the GP’s ability to encourage and support them in their care more than on therapeutic solutions [8]. A literature review identified three main priorities in healthcare delivery to migrants: improved communication with patients, continuity of care (with education for migrants and refugees about the healthcare system, easy access to health facilities, integration of medical appointments, and collaboration with institutions) and trust between patients and care providers [40]. In mainstream practice, GPs’ attitudes play an important role in ensuring migrants’ adherence to and navigation of the healthcare system. With a patient-centered approach, providing culturally adapted care and assisting with building patients’ health systems literacy can enhance care experiences [22]. When GPs care for underserved populations, they develop specific skills to improve physician‒patient relationships and meet their patients’ health-related needs [41].

This raises the question of what resources are available to outpatient GPs to enable them to treat these patients efficiently. Organizational innovations (e.g., health mediation, direct access to common ambulatory general practice, etc.) are likely to improve the integration of migrants into the common primary care system. The use of professional interpreters has already demonstrated benefits in terms of health service quality and reduced costs associated with misunderstandings for patients and practitioners [42, 43]. Health and cultural mediation are developing in primary healthcare. If these interventions seem to improve care experiences, increase understanding and reduce healthcare costs [44–46], then studies are needed to evaluate mediation in various contexts, including primary and transitional care. Innovative organizations can provide direct access to mainstream general practice for uninsured patients. A study explored the experiences of patients who benefited from an ambulatory PASS network in Marseille and who obtained immediate access to common primary care (general practitioners, gynecologists and/or dentists) in the city before their health insurance rights were established. They felt more socially integrated through consideration by healthcare providers. Most still identified the GP they encountered during the PDV as their referent doctor once their health insurance rights were established [47]. Further studies evaluating the impact of these organizations should suggest skills to improve migrants’ quality of care.

### Limitations

Our study was conducted only in France, but all countries have healthcare facilities for uninsured migrants that represent different approaches to tailored services. These results could be discussed through the logic of transition from tailored to non-tailored services. Males represented most of our sample, as females tend to face specific barriers to accessing healthcare (e.g., exposure to sexual violence for women who changed their use of care and their relationships with healthcare providers, specific barriers in sexual health prevention or maternity care, which could necessitate the development of specific referrals and empowerment strategies, etc.) [19, 37, 48]. The qualitative approach chosen for this study provided us with a deeper understanding of patients’ specific experiences and strategies for seeking care but did not aim to achieve generalizable outcomes. Quantitative studies should evaluate the effects of various strategies for referral in primary care after patients receive care in an SPHC. Future research could also further analyze these issues with a larger, more diversified sample, including migrants and vulnerable citizens with the nationality of the country studied.

## Conclusion

The WHO has noted that UHC presupposes an efficient primary care system capable of integrating vulnerable migrants who are currently using services at SPHCs [49]. Our study provides initial findings to explore this necessary transition. Our results suggest that migrants’ adherence to common ambulatory general practice after receiving care in SPHCs should not only depend on general practitioners’ attitudes, skills, and organization but that SPHCs could also be improved by referral protocols involving health mediation, especially for vulnerable migrants who develop strong attachments to SPHCs. These issues should be examined through quantitative studies evaluating the impact of different strategies for referring migrants to common ambulatory general practice after they receive SPHC services.

### Supplementary Information

Supplementary Material 1. COREQ 32 checklist.

Supplementary Material 2. Interview guide.

## Data Availability

The datasets used and/or analyses of the current study are available from the corresponding author upon reasonable request.

## Abbreviations

CNIL: French Data Protection Authority
PASS: Permanence d’accès aux soins de santé (French)
SPHC: Specific primary healthcare facility
WHO: World Health Organization
