# Supplementary material for: Care-seeking strategies of migrants during the transition from a specific primary healthcare facility for uncovered individuals to common ambulatory general practice: A French qualitative study
Source: BMC Public Health. 2024 Jun 10;24:1552. doi: 10.1186/s12889-024-19048-x (PMC11163736; doi:10.1186/s12889-024-19048-x)
Supplement: Supplementary file 1 — Supplementary Material 1. COREQ 32 checklist. [file 12889_2024_19048_MOESM1_ESM.docx]

Supplementary material - COREQ chelcklist

| Domain 1 : research team and reflexivity |  |  |
| --- | --- | --- |
| 1. Interviewer/facilitator | Which author conducted the interview or focus group ? | FALL Marie and DESRUES Anne conducted the interviews. |
| 1. Credentials | What were the researcher’s credentials ? | FALL Marie was resident in general practice, MD student.  DESRUES Anne has a master thesis in sociology and was working as investigator for Aix-Marseille University  This work was co-directed by Dr JEGO Maeva (MD-PHD, senior registrar at Aix-Marseille University)  And Dr KHOUANI Jeremy (MD, PHD student, general practitioner, senior registrar at Aix-Marseille University). |
| 1. Occupation | What was their occupation at the time of the study ? | FALL Marie : MD student, resident in general practice.  DESRUES Anne : Working as interviewer for several studies linked to MULTIPASS study (quantitative and qualitative phases), and INCIDAVI study (exploring in quantitative and qualitative phases sexual violences in France on women asylum seekers).  JEGO Maeva : general practitioner (multiprofessionnal Health Center « Peyssonnel », 13003, Marseille), senior registrar at Aix-Marseille University (department of general practice). She was used to receive and care people who beneficited from PASS.  KHOUANI Jeremy : general practitioner (multiprofessionnal Health Center « Peyssonnel », 13003, Marseille), senior registrar at Aix-Marseille University (department of general practice).He was used to receive and care people who beneficited from PASS. |
| 1. Gender | Was the researcher male or female ? | FALL Marie : female  DESRUES Anne : female  JEGO Maeva : female  KHOUANI Jérémy : male |
| 1. Experience and training relationship with participants | What experience or training did the researcher have ? | FALL Marie : read qualitative methods’ books before beginning the research. She received personnal training by the co-director of this research (JEGO Maeva).  DESRUES Anne : already worked on  several qualitative  sociological studies as an independent researcher and as a member of a public policy consulting firm.  Her master thesis was also a qualitative study on gender role.  JEGO Maeva already performed qualitative studies for master thesis and MD thesis. She directed 17 qualitative research for MD student in general practice. She followed a two days course about qualitative research and already gave courses to students about qualitative research. She is member of the GROUM-F (Groupe Universitaire de Recherche Qualitative Médicale Francophone) and followed then 4 workshops about qualitative research. She is expert for the CNGE (collège national des généralistes enseignants) for trainings in qualitative research. |
| 1. Relationship established | Was a relationship established prior to study commencement ? | Yes. |
| 1. Participant knowledge of the interviewer | What did the participants know about the researcher ? | The participants knew the interviewer' education diploma and background and were briefly explained the qualitative methodology purpose |
| 1. Interviewer characteristics | What characteristics were reported about the interviewer | M.F., resident in general practice, and A.D., sociologist |
| Domain 2 : study design |  |  |
| 1. Methodological orientation and theory | What methodological orientation was stated to underpin the study ? | Grounded theory approach |
| 1. Sampling | How were participant selected ? | Purposive method (purposefull variation sampling) |
| 1. Method of approach | How were participants approached ? | Mostly by telephone to make the contact. Then the interviews were conducted in face to face or by phone (only 1 by phone) |
| 1. Sample size | How many participants were in the study ? | 12 |
| 1. Non-participation setting | How many people refused to participate or dropped out ? | 6 |
| 1. Setting of data collection | Where was the data collected ? | Mostly on an office at the PASS service, or by phone. |
| 1. Presence of non-participants | Was anyone else present besides the participants and researchers ? | No |
| 1. Description of sample data collection | What are the important characteristics of the sample ? | 12 patients were interviewed between January to april 2022.  The interviews lasted for approximately 56 minutes (24 to 80 minutes). The patients were 22 to 65 years-old (median age 40 years). Almost all the interviews (11) were passed in face to face. Eight patients on 12 were men. Most of the patients were from sub-saharan Africa (7), 3 were from Maghreb and 2 from Est Europa. Patients were mostly on irregular situation (8 on 12), and beneficiated from free medical aid (state medical aid) |
| 1. Interview guide | Were questions, prompts, guides provided by the authors ? Was it pilot tested ? | The questions were prompts.  The guides are provided in annex.  The guide was pilot tested (1 pilot test) |
| 1. Repeat interviews | Were repeat interviews carried out ? | No |
| 1. Audio/visual recording | Did the research use audio or visual recording to collect the data ? | Yes (Audio recording) |
| 1. Field notes | Were field notes made during and/or after the interview or focus group ? | Yes |
| 1. Duration | What was the duration of the interviews or focus group ? | 56 minutes |
| 1. Data saturation | Was data saturation discussed ? | Yes |
| 1. Transcripts returned | Were transcripts returned to participants for comment and/or correction ? | No (due to protocol and confidential policy). |
| Domain 3 : analysis and findings |  |  |
| 1. Number of data coders | How many data coders coded the data ? | 2 (FALL Marie and DESRUES Anne) supervised by JEGO Maeva. |
| 1. Description of coding tree | Did authors provide a description of the coding tree ? | Yes |
| 1. Derivation of themes | Were themes identified in advance or derived from the data ? | Derived from the data |
| 1. Software | What software, if applicable, was used to manage the data ? | N Vivo V11 |
| 1. Participant checking reporting | Did participants provide feedback on the findings | No |
| 1. Quotations presented | Were participants quotations presented to illustrate the themes / findings ? Was each quotation identified ? | Yes.  Yes |
| 1. Data and findings consistent | Was there consistency between the data presented and the findings ? | Yes |
| 1. Clarity of major themes | Were major themes clearly presented in the findings ? | Yes |
| 1. Clarity of minor themes | Is there a descrption of diverse cases or discussion of minor themes ? | Yes |
