# Supplementary material for: Care-seeking strategies of migrants during the transition from a specific primary healthcare facility for uncovered individuals to common ambulatory general practice: A French qualitative study
Source: BMC Public Health. 2024 Jun 10;24:1552. doi: 10.1186/s12889-024-19048-x (PMC11163736; doi:10.1186/s12889-024-19048-x)
Supplement: Supplementary file 2 — Supplementary Material 2. Interview guide. [file 12889_2024_19048_MOESM2_ESM.docx]

# Interview guide (final version)

**Introduction to the interview:**

"Hello, thank you for agreeing to take part in this interview. My name is (name of interviewer here). I'm a health student / researcher and we're meeting today to explore the outpatient care experiences of patients who have been followed up by the AP-HM Hospital PASS. There is no right or wrong answer, it's all about exploring your own experiences and feelings. No value judgements will be made. You are free to express whatever you wish. The interview is confidential and anonymous.

Before we start:

- Have you signed the consent form?

- Do you have any questions before we start?

**Interview procedure**

1) You recently visited a general practitioner. Can you tell me about this visit?

2) What do you expect from your family doctor? Relaunch: What do you mean by a general practitioner or family doctor?

3) What are your expectations and fears about choosing a GP?

4) How did you meet your GP? Would you have liked to meet him in a different way? And if so, how?

5) What was your experience about your health care at the PASS? Relaunch: What does the PASS mean to you?

6) How did you receive health care in France before coming to PASS?

7) How did your health care in the PASS end? What was your experience about it?

8) How did you find the transition from the PASS hospital to outpatient care? Relaunch: What was your experience of being referred to the GP you consulted? Were there any things that should have been done differently? What did the transition from PASS to GP mean to you?

9) Finally, what do you think of your care (or care pathway)? would you have liked it any other way?

Is there anything you would like to add that we haven't mentioned?

Thank you for your time.
